# Synergistic effects of multiple enzymes from industrial *Aspergillus niger* strain O1 on starch saccharification

**DOI:** 10.1186/s13068-021-02074-x

**Published:** 2021-11-27

**Authors:** Wenzhu Guo, Jianhua Yang, Tianchen Huang, Dandan Liu, Qian Liu, Jingen Li, Wenliang Sun, Xingji Wang, Leilei Zhu, Chaoguang Tian

**Affiliations:** 1 Key Laboratory of Systems Microbial Biotechnology, Tianjin Institute of Industrial Biotechnology, Chinese Academy of Sciences, Tianjin, 300308 China; 2 National Technology Innovation Center of Synthetic Biology, Tianjin, 300308 China; 3 Longda Biotechnology Inc, Shandong, 276400 China

**Keywords:** *Aspergillus niger*, Glucoamylase, α-Amylase, Starch saccharification, Synergistic effects

## Abstract

**Background:**

Starch is one of the most important renewable polysaccharides in nature for production of bio-ethanol. The starch saccharification step facilitates the depolymerization of starch to yield glucose for biofuels production. The filamentous fungus *Aspergillus niger* (*A. niger*) is the most used microbial cell factory for production of the commercial glucoamylase. However, the role of each component in glucoamylases cocktail of *A. niger* O1 for starch saccharification remains unclear except glucoamylase.

**Results:**

In this study, we identified the key enzymes contributing to the starch saccharification process are glucoamylase, α-amylase and acid α-amylase out of 29 glycoside hydrolases from the 6-day fermentation products of *A. niger* O1. Through the synergistic study of the multienzymes for the starch saccharification in vitro, we found that increasing the amount of α-amylase by 5-10 times enhanced the efficiency of starch saccharification by 14.2-23.2%. Overexpression of acid α-amylase in strain O1 in vivo increased the total glucoamylase activity of O1 cultures by 15.0%.

**Conclusions:**

Our study clarifies the synergistic effects among the components of glucoamylases cocktail, and provides an effective approach to optimize the profile of saccharifying enzymes of strain O1 for improving the total glucoamylase activity.

**Supplementary Information:**

The online version contains supplementary material available at 10.1186/s13068-021-02074-x.

## Background

Starch is one of the most important renewable polysaccharides for the production of bio-ethanol [1, 2]. Starch is composed of two α-glucan polymers, amylose and amylopectin [3]. Amylose is a linear chain of α-glucose linked by α-1,4-glycosidic linkages, while amylopectin is a branched macromolecule linked by α-1,4-glycosidic linkages and α-1,6-glycosidic linkages [4–6]. Cost-effective conversion of raw starch to biofuels requires efficient starch-hydrolyzing enzyme system.

*Aspergillus niger* (*A. niger*) strains can secrete a broad range of hydrolytic enzymes required for degradation of polymeric substances (e.g. starch) to release nutrients [7, 8]. The filamentous fungus *A. niger* is used as microbial cell factory for production of lignocellulolytic enzymes and amylolytic enzymes [9–12]. Amylolytic enzymes that hydrolyze the glycosidic linkages in α-glucans belong to three glycoside hydrolases (GHs) families: GH13 (α-amylases), GH14 (β-amylases) and GH15 (glucoamylases) [5, 13, 14]. α-Amylases and glucoamylases are widely used in starch conversion [15]. α-Amylases can randomly cleave the internal α-1,4-glycosidic linkages in starch to generate maltose and malto-oligosaccharides [16–18]. Glucoamylase (glucan 1,4-α-glucosidase) is a typical exo-acting enzyme that hydrolyzes starch completely into glucose from the non-reducing ends [12, 19–21]. Starch liquefaction by thermostable α-amylase and saccharification mainly by glucoamylase are sequentially carried out to produce glucose from starch for the production of bio-ethanol in industry [22, 23]. Therefore, glucoamylase is one of the most needed industrial enzymes in biofuel industry [24].

Commercial glucoamylase is usually produced by fermentation process of *A. niger*. Commercial glucoamylase used in industrial production is a glucoamylases cocktail (multi-enzyme system). Moreover, it has been reported that improving the expression of some specific enzymes can enhance the total enzyme activity of the glucoamylase enzyme system. Synergistic action of α-amylase and glucoamylase on hydrolysis of starch in vitro showed that at the early stage of reaction, α-amylase accelerated the rate of formation of glucose by supplying newly formed non-reducing ends of starch molecules [25]. By overexpressing *amyA* and *glaA*, An et al. increased the total extracellular glucoamylase activity of *A. niger* by 44.1% with 1% casein phosphopeptides added to the fermentation medium [22]. Parahar et al. achieved one-step starch saccharification by engineering a chimeric acid-stable α-amylase-glucoamylase in *E. coli* [26]. Wang et al. provided evidence for synergistic effect of α-amylase and glucoamylase on alcoholic fermentation for Chinese baijiu production [27]. The activities of total glucoamylase in red and white koji were similar. The α-amylase activity in white koji was 100-fold higher than that in red koji and saccharification in red koji is lower than that in white koji due to low α-amylase activity. They speculated that increasing α-amylase production enhance red koji activity in saccharification [28]. However, except for the main glucoamylase, the contribution of each component within the multiple starch degradation enzymes composing glucoamylase cocktails remains unclear. Therefore, it is important to study their synergistic effects on starch saccharification for further improvement of glucoamylase cocktail performance to achieve efficient glucose production.

In this study, we purified and characterized the main components of the enzymes from the fermentation product of an industrial glucoamylase-producing strain *A. niger* O1. The contribution of each component in the starch saccharification was investigated by combination of synergistic study of glucoamylase system in vitro and genetic manipulation in vivo. Our study clarifies the synergistic effects among the components of glucoamylases cocktail, and provides an effective approach to optimize the total activity of glucoamylases cocktail produced by industrial fungal strain O1.

## Results

### Profile of the secreted proteins of *A. niger* strain O1

**Fig. 1:**
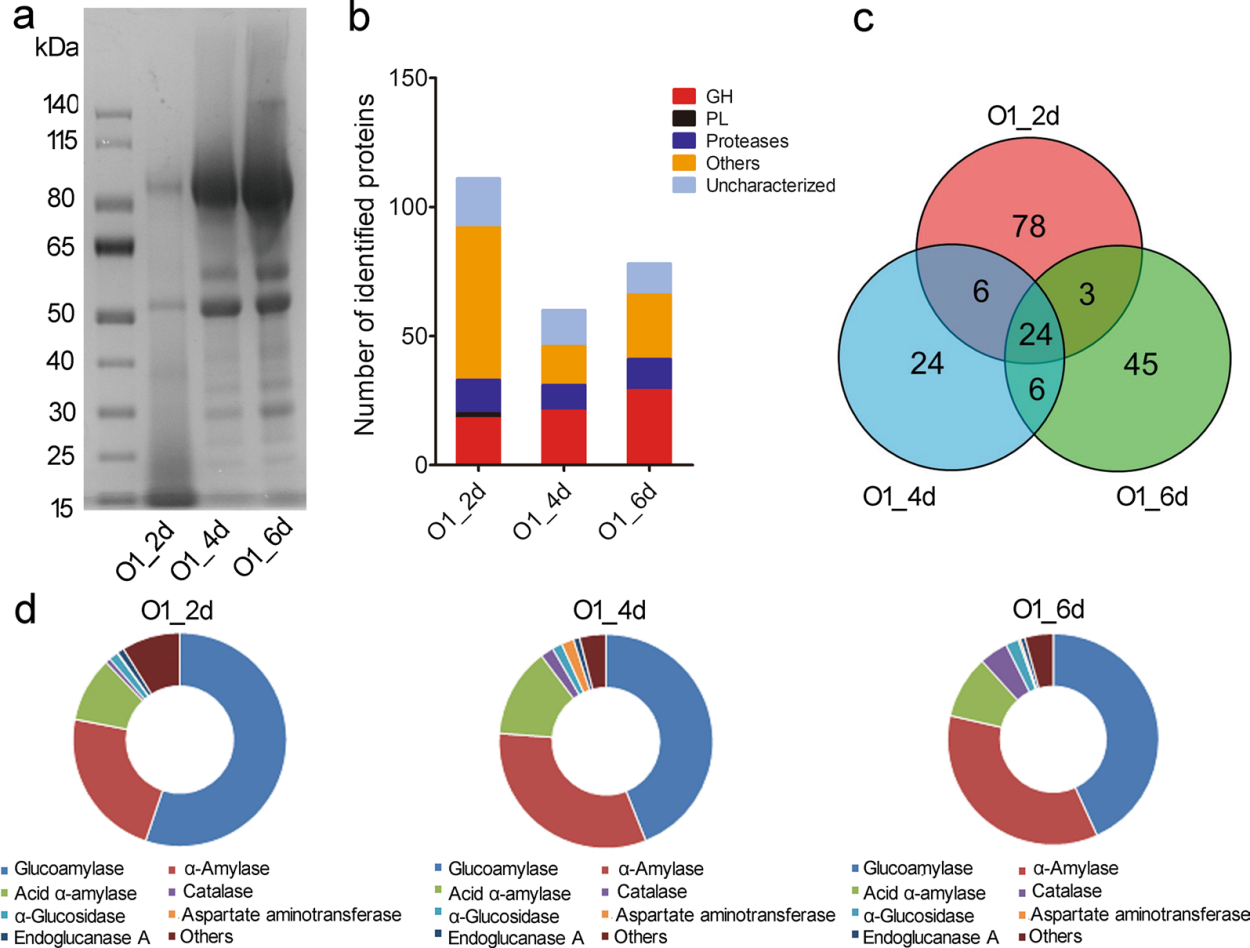
Comparative secretome analysis of the *A. niger* O1 in shake-flask fermentation. **a** SDS-PAGE analysis of secreted proteins in 2 days, 4 days, 6 days supernatants of O1 fermentation cultures. **b** Category of identified proteins in 2 days, 4 days, 6 days supernatants of O1 fermentation cultures. *GH* glycoside hydrolase; *PL* polysaccharide lyase. **c** Venn diagram of the comparison of identified proteins in 2 days, 4 days, 6 days supernatants of O1 fermentation cultures. **d** A schematic diagram that roughly displayed the detailed varieties and relative amount of secreted protein by *A. niger* O1 after 2 days, 4 days, 6 days cultivation

The filtered supernatants of 2-day (2 d), 4-day (4 d) and 6-day (6 d) shake-flask cultures of *A. niger* strain O1 were analyzed by SDS-PAGE (Fig. 1a). To explore the changes of secreted proteins in fermentation process, filtered supernatants were analyzed using liquid chromatography combined with tandem mass spectrometry (LC-MS/MS). Label-free quantitative proteomics approach was used to study the enzyme profile in the fermentation cultures. As a result, 111, 60 and 78 proteins were identified from the 2 days, 4 days and 6 days fermentation supernatants, respectively (Fig. 1b). There were 19, 20 and 29 glycoside hydrolases detected in 2-day, 4-day and 6-day fermentation supernatants and the ratio of glycoside hydrolases in identified proteins are 17.1%, 33.3% and 37.2% in 2-day, 4-day, 6-day fermentation cultures, respectively (Fig. 1b; Additional file 1: Fig. S1). 24 proteins were consistently present in 2-day, 4-day and 6-day supernatants in fermentation process (Fig. 1c). 13 proteins among these 24 proteins belong to glycoside hydrolase families. Glucoamylase of GH15, α-amylase and acid α-amylase of GH13 are the most abundant proteins in 2-day, 4-day and 6-day fermentation cultures, accounting for most part of the total proteins (Fig. 1d). Other proteins such as catalase, aspartate aminotransferase, α-glucosidase and endoglucanase also take a certain proportion of secretome.

### Isolation, purification, and identification of enzymes related to starch saccharification from secreted proteins of *A. niger* strain O1

**Fig. 2:**
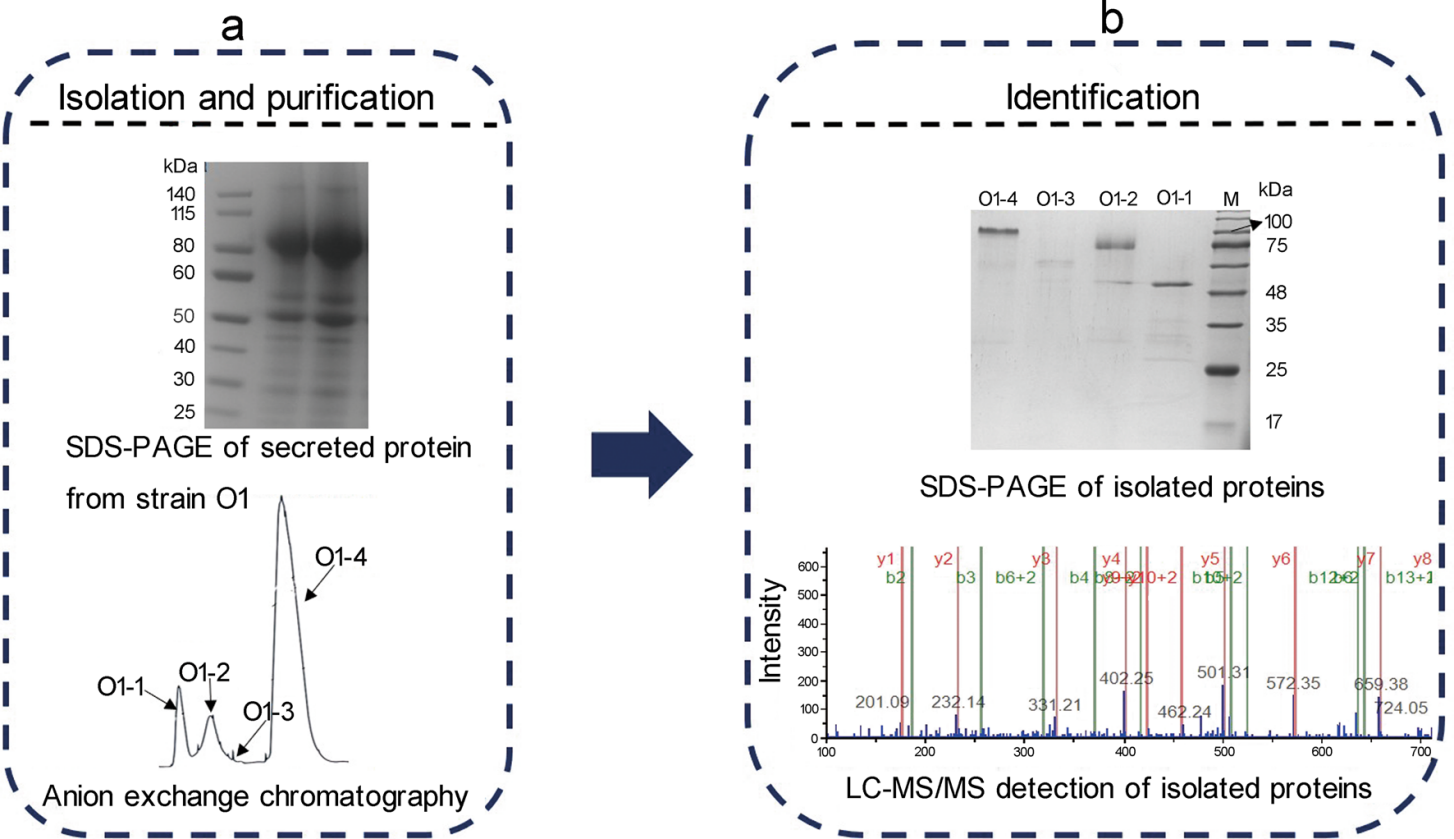
The processes of isolation and identification of starch saccharification-related enzymes from secreted proteins of *A. niger* O1.** a** (up) SDS-PAGE of the supernatants of the industrial glucoamylase-producing *A. niger* O1 after 6 days cultivation. (down) Isolation and purification of enzymes related to starch saccharification from secreted proteins of O1 using anion exchange chromatography. **b** (up) SDS-PAGE of isolated enzymes related to starch saccharification from secreted proteins of O1. Four lanes and M respectively represent O1-1, O1-2, O1-3, O1-4 and protein ladder. (down) LC-MS/MS detection of isolated proteins. O1-1 is neutral α-amylase, O1-3 is acid α-amylase, and O1-2 and O1-4 are glucoamylases with different glycosylation level

The procedure of isolation and identification of enzymes contributing to the starch saccharification from the 6-day supernatants of the *A. niger* strain O1 is shown in Fig. 2. Through the isolation and purification of the enzymes by anion exchange chromatography, four main enzyme components (O1-1, O1-2, O1-3, and O1-4) were isolated with the ratio of protein concentration of 9.9:10:0.1:80. The protein concentration was monitored according to the absorbance at UV 280 nm (Fig. 2a). Therefore, the quantity of O1-1, O1-2, O1-3, and O1-4 was deemed to be 9.9%, 10%, 0.1%, and 80% of the total protein, respectively. The molecular weight of O1-1, O1-2, O1-3, and O1-4 are about 50, 75, 63, and 100 kDa as shown on SDS-PAGE, respectively (Fig. 2b). Through LC-MS/MS detection, O1-1 and O1-3 were identified as neutral α-amylase (UniProt: A2QL05) and acid α-amylase (UniProt: A2QW02), respectively; O1-2 and O1-4 were confirmed to be glucoamylase (UniProt: A2QHE1) (Fig. 2b; Additional file 2). The molecular weight of O1-4 is larger than O1-2, which might be caused by the different glycosylation level. After deglycosylation of O1-2 and O1-4 using PNGase F, the sizes of both proteins reduced, but still different as shown in Additional file 5: Fig. S2. This may be due to different kinds of O-linked glycosylation that cannot be digested by PNGase F [29].

### Characterization of each component function of glucoamylases cocktail secreted by *A. niger* strain O1

**Fig. 3:**
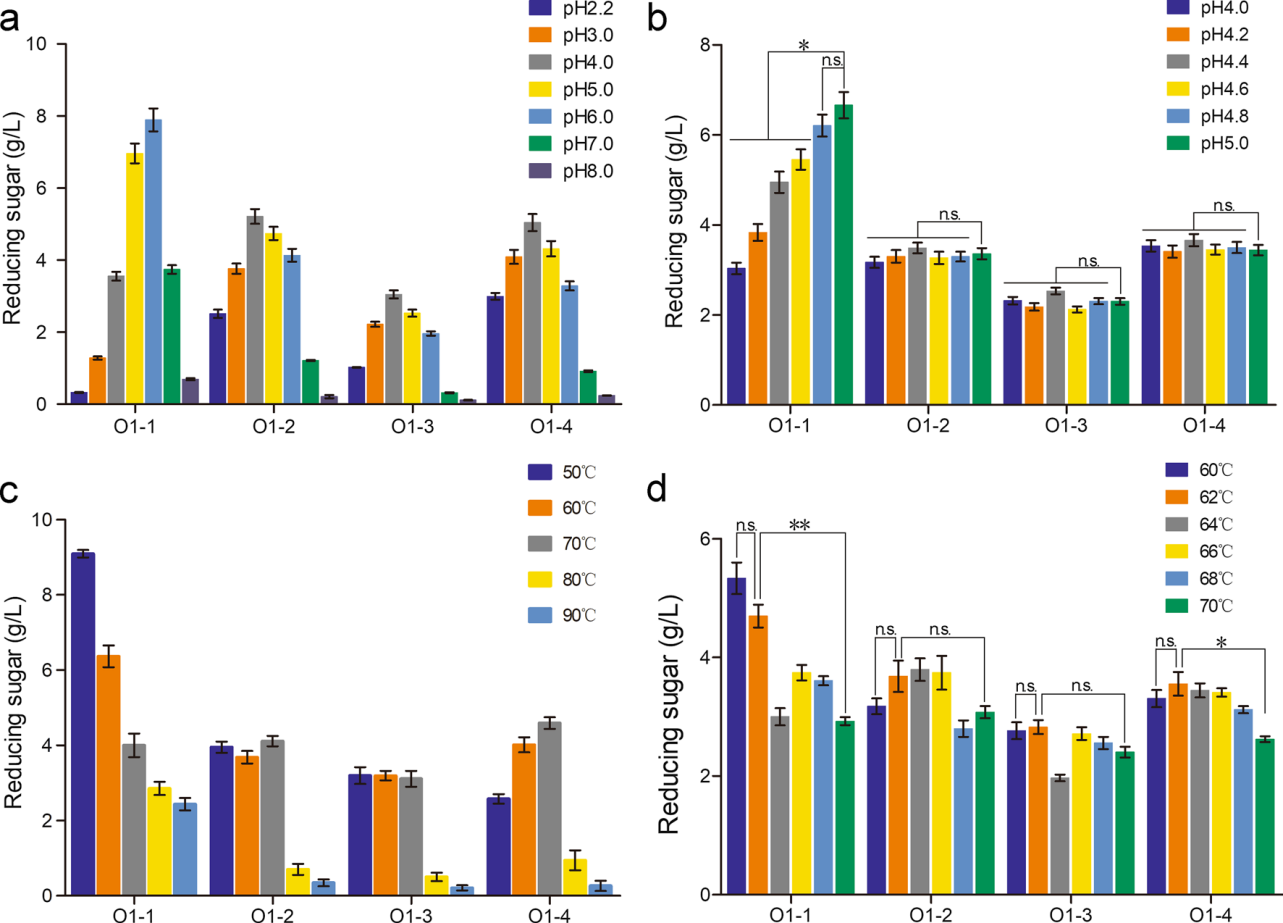
Characterization of each component function of glucoamylases cocktail secreted from *A. niger* O1. **a** and **b** The optimal pH of different starch saccharification-related enzymes. **c** and **d** The optimal reaction temperature of different starch saccharification-related enzymes. O1-1: neutral α-amylase, O1-2: low-glycosylated glucoamylase, O1-3: acid α-amylase, and O1-4: high-glycosylated glucoamylase. Values are means ± SD (*n* = 3 repeats). **P < 0.01 (Student’s *t*-test), *P < 0.05 (Student’s *t*-test), *n.s.* not significant

In order to optimize the reaction conditions of the starch saccharification, varied reaction pH and temperatures of each identified enzyme were investigated. As shown in Fig. 3a and b, the optimal pH of the O1-1 (neutral α-amylase), O1-2 (low-glycosylated glucoamylase), O1-3 (acid α-amylase), and O1-4 (high-glycosylated glucoamylase) are 6.0, 4.4, 4.4 and 4.4, respectively. For O1-2, O1-3 and O1-4, the activity at the pH from 4.0 to 5.0 change slightly, but O1-1 shows higher enzyme activity at near neutral pH (6.0). Therefore, the pH for further synergistic study of saccharification enzyme system was set up at 5.0. O1-1, O1-2, O1-3 and O1-4 show the highest activity at 50 ℃, 64 ℃, 62 ℃ and 62 ℃, respectively (Fig. 3c and d). O1-4 (glucoamylase) is the most important enzyme in starch scarification, thus the optimal temperature for further study of synergistic degradation of starch was set as the optimal temperature of O1-4 (62 °C). Considering the other three enzymes showing no obvious decrease in activity at 62 °C, it was finally determined that the reaction temperature of the synergistic enzyme system is 62 °C and the reaction pH is 5.0.

### Synergistic effect of each enzyme component in glucoamylases cocktail on starch saccharification

**Fig. 4:**
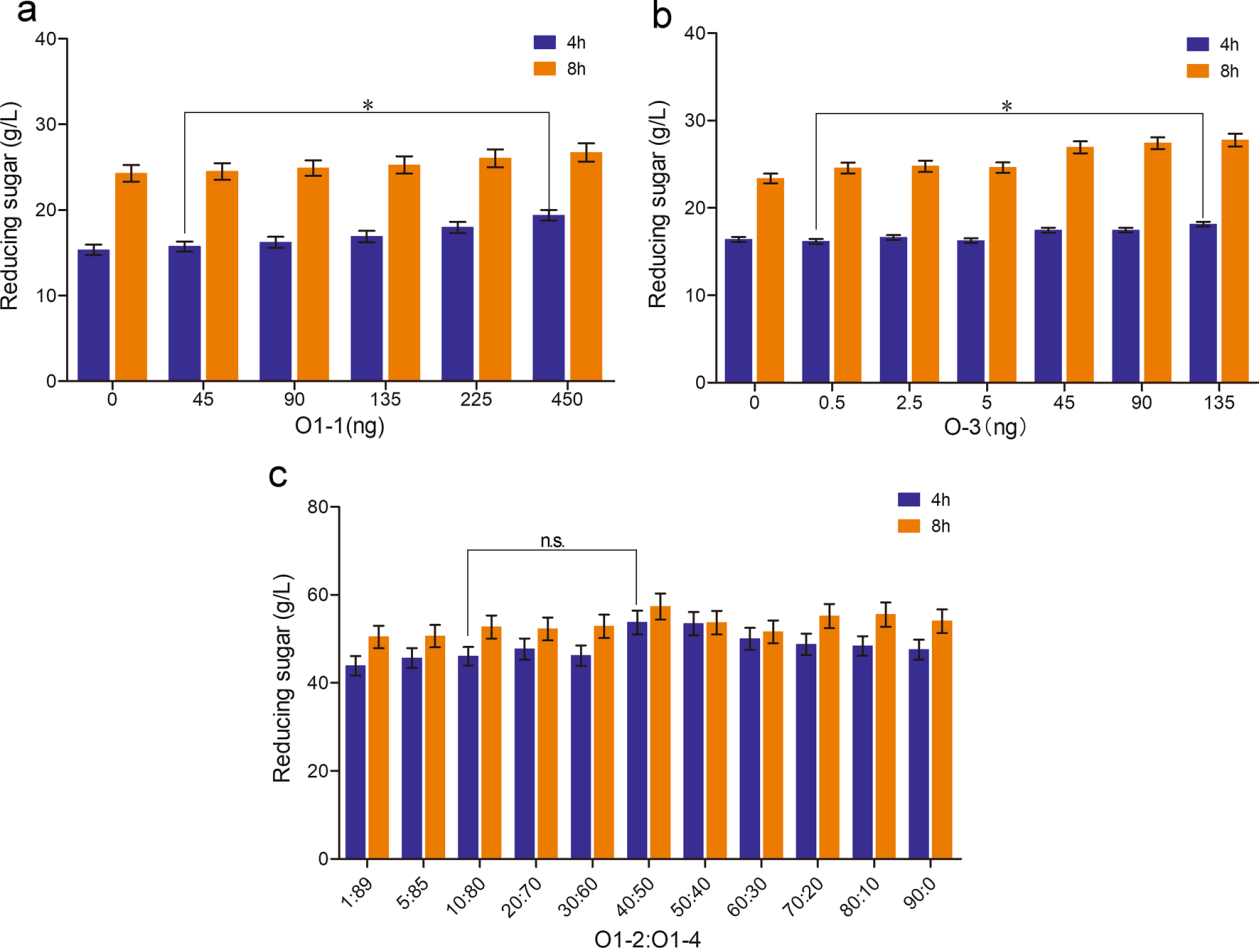
Synergistic effects of each enzyme component in glucoamylases cocktail on starch saccharification. **a** The effect of O1-1 neutral α-amylase on glucoamylases cocktail for starch saccharification. **b** The effect of O1-3 acid α-amylase on glucoamylases cocktail for starch saccharification. **c** The effect of the ratio of two glucoamylases on glucoamylases cocktail for starch saccharification. Values are means ± SD (*n* = 3 repeats). *P < 0.05 (Student’s *t*-test), *n.s.* not significant

The proportion of different enzymes in the synergistic system was adjusted and optimized based on the proportion in the secreted protein of strain O1 (O1-1:O1-2:O1-3:O1-4 = 9.9:10:0.1:80). As shown in Fig. 4a, increasing the amount of α-amylase by 5-10 times in vitro led to boosted yield of reducing sugar by 14.2-23.2%. As shown in Fig. 4b, when less than 1% acid α-amylase was added, the produced reducing sugar increased not more than 3.0% compared to the original enzyme ratio in glucoamylases cocktail (0.1% acid α-amylase). When the supplemented acid α-amylase was greater than 8%, the yield of reducing sugar increased obviously. As shown in Fig. 4c, the optimal ratio of O1-2:O1-4 is 40:50. The reducing sugar production increased by 8.8-16.6% when changing the ratio of O1-2:O1-4 from 10:80 to 40:50.

### The effects of overexpression and knock out of acid α-amylase (AA) gene on the total glucoamylase activity of secreted proteins from O1

**Fig. 5:**
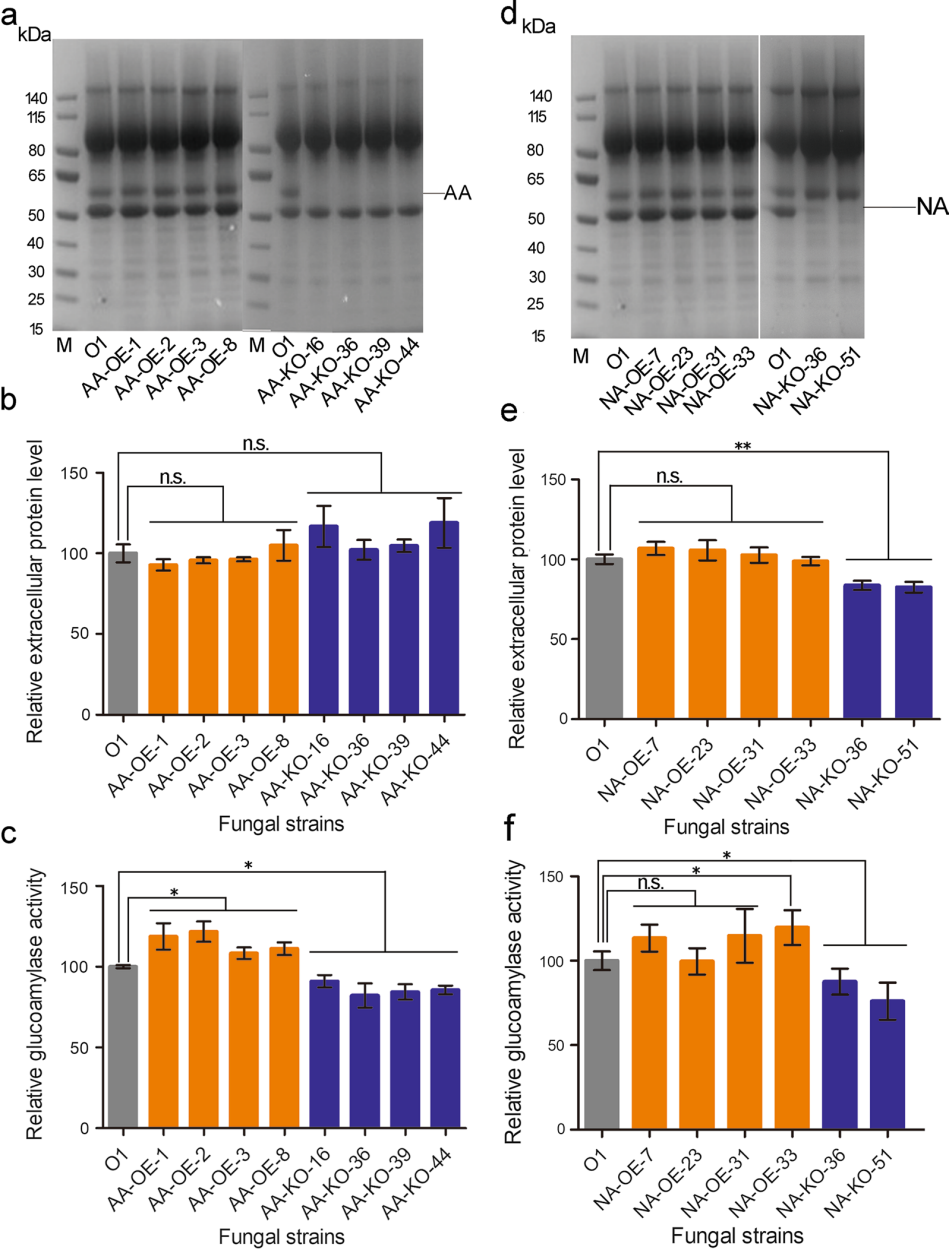
The effects of overexpression and knock out of acid α-amylase (AA) and α-amylase (NA) genes on protein expression level and total glucoamylase activity. **a** SDS-PAGE analysis of secreted proteins in 6-day supernatants of fermentation cultures of *A. niger* O1, AA-OE and AA-KO strains. **b** Extracellular protein level detection in 6-day supernatants of fermentation cultures of *A. niger* O1, AA-OE and AA-KO strains. Relative extracellular protein level = Extracellular protein level of engineered strain to that of O1. **c** The total glucoamylase activity assay in 6-day supernatants of fermentation cultures of *A. niger* O1, AA-OE and AA-KO strains. Relative glucoamylase activity = glucoamylase activity of engineered strain to that of O1. **d** SDS-PAGE analysis of secreted proteins in 6 days supernatants of fermentation cultures of *A. niger* O1, NA-OE and NA-KO strains. **e** Extracellular protein level detection in 6 days supernatants of fermentation cultures of *A. niger* O1, NA-OE and NA-KO strains. Relative extracellular protein level = extracellular protein level of engineered strain to that of O1. **f** The total glucoamylase activity assay in 6 days supernatants of fermentation cultures of *A. niger* O1, NA-OE and NA-KO strains. The total glucoamylase activities of transformants and O1 were measured using the same amount of total protein in supernatants. Relative glucoamylase activity = glucoamylase activity of engineered strain to that of O1. Values are means ± SD (*n* = 3 repeats). **P < 0.01 (Student’s *t*-test), *P < 0.05 (Student’s *t*-test), *n.s.* not significant

Based on the results of synergistic study, we designed a binary vector for overexpression of acid α-amylase gene. The donor DNA and sgRNA cassettes of acid α-amylase (O1-3) gene were designed and constructed for CRISPR-Cas9 gene editing system to knock out acid α-amylase gene. After protoplasts transformation, four homozygous AA-OE (overexpression) and four homozygous AA-KO (knock out) transformants were obtained and identified by diagnostic PCR. The supernatants of the 6-day cultures were assayed by SDS-PAGE which shows improved acid α-amylase expression in AA-OE strains and disappeared acid α-amylase in AA-KO strains compared to host strain O1 with loading the same amount of total protein (Fig. 5a). This was also verified by the real-time PCR (Additional file 6: Fig. S3a). The concentration of total extracellular protein was detected using the supernatants of 6-day fermentation cultures. The results indicate that AA-OE and AA-KO strains have similar extracellular protein levels compared with the host stain O1 (Fig. 5b). The total glucoamylase activity of the filtered supernatants of transformants and O1 were measured. The AA-OE strains (AA-OE-1, AA-OE-2, AA-OE-3, AA-OE-8) exhibit significantly higher glucoamylase activity than O1 by 18.7%, 21.7%, 8.3%, and 11.1%, respectively (Fig. 5c). The glucoamylase activity of the AA-KO transformants AA-KO-16, AA-KO-36, AA-KO-39 and AA-KO-44 were significantly reduced to 90.9%, 82.1%, 84.3% and 85.5% of that of O1, respectively (Fig. 5c).

### The effects of overexpression and knock out of neutral α-amylase (NA) gene on the total glucoamylase activity of secreted proteins from O1

The experiments for neutral α-amylase overexpression and knock out were similarly designed as the acid α-amylase overexpression and knock out. Overexpression binary vector and donor DNA, sgRNAs were constructed, respectively, for NA-OE (overexpression) and NA-KO (knock out). After protoplasts transformation, four homozygous NA-OE and two homozygous NA-KO transformants were obtained and identified using diagnostic PCR. The 6-day culture supernatants were assayed by SDS-PAGE on which NA-OE strains display thicker neutral α-amylase band and NA-KO strains show disappeared neutral α-amylase band compared to the host strain O1 with loading the same amount of total protein (Fig. 5d). This was also verified by the real-time PCR (Additional file 6: Fig. S3b). The concentration of total extracellular proteins in 6 days fermentation cultures was also detected. The protein levels of NA-OE-7, NA-OE-23 and NA-OE-31 slightly increased by 6.7%, 5.6% and 2.6%, and the protein level of NA-OE-33 slightly decreased by 1.3% than O1 (Fig. 5e). The protein levels of two NA-KO strains NA-KO-36 and NA-KO-51 significantly decreased by 16.4% and 17.5% compared with that of the host strain O1 (Fig. 5e). We also analyzed the total glucoamylase activity of supernatants of four NA-OE strains, two NA-KO strains and O1. Enzymatic assay results exhibit that three of four NA-OE strains have improved enzymatic activity by 13.5%, 14.8%, 19.6% and NA-OE-23 has almost the same enzymatic activity as O1 (Fig. 5f); whereas, the glucoamylase activity of two NA-KO strains significantly reduced to 87.6% and 76.0% of that of the host strain O1 (Fig. 5f).

## Discussion

Starch saccharification is the important step for production of high value-added downstream industry products, such as bio-ethanol, amino acids, pharmaceuticals [15, 30, 31]. The efficiency of saccharification process depends on the total glucoamylase activity. Glucoamylase used in industry is not a single enzyme, but a mixture of various starch-degrading enzymes. All enzymes playing roles in starch saccharification constitute a glucoamylase enzyme system in which the glucoamylase is the major functional enzyme. However, the exact contributions of each of these enzymes during the starch saccharification working with glucoamylase remain unclear.

In our study, we tried to dig up more enzymes associated with starch saccharification from industrial strain *A. niger* O1 and study their secreted proteome profiles during fermentation process for glucoamylases system production. We found that glycoside hydrolases detected in secreted proteome increased from 2 to 6 days fermentation cultures during the whole fermentation process (Fig. 1b). The three proteins accounting for the highest amount (glucoamylase, α-amylase and acid α-amylase) belong to glycoside hydrolase families and are involved in starch saccharification (Fig. 1d). The other two enzymes (catalase and aspartate aminotransferase) in fermentation cultures might be important for growth of strain O1, but not for starch degradation. Additionally, endoglucanase A is an enzyme belonging to GH12 with the annotation of cellulase activity. Although α-glucosidase is a starch-degrading glycoside hydrolase in fermentation cultures, it is undesirable for starch saccharification because of its transglycosylation activity for producing unfermentable sugars [32–34]. Other glycoside hydrolases detected in fermentation supernatants are related to the degradation of other polysaccharides not starch (Additional file 1: Fig. S1). So glucoamylase, α-amylase and acid α-amylase are the main components in glucoamylase enzyme system of industrial strain O1 for starch saccharification.

Four main protein components were isolated from the supernatants of the 6-day fermentation cultures and named as O1-1, O1-2, O1-3 and O1-4. LC-MS/MS detection and enzymatic activity assay confirmed the specific ingredients of four isolated proteins: neutral α-amylase (O1-1), low-glycosylated glucoamylase (O1-2), acid α-amylase (O1-3) and high-glycosylated glucoamylase (O1-4) (Fig. 2a and b). Characterization of four enzymes confirmed the optimal temperature and the optimal pH: O1-1 (50 ℃, pH 5.0), O1-2 (64 ℃, pH 4.4), O1-3 (62 ℃, pH 4.4), O1-4 (62 ℃, pH 4.4), respectively (Fig. 3a-d). These results provided the optimal reaction conditions for the glucoamylase enzyme system of strain O1 (62 ℃, pH 5.0). The in vitro synergistic effects analysis showed that the 10 times increased neutral α-amylase or acid α-amylase improved the saccharification efficiency of soluble starch by 8.0-23.2% (Fig. 4a, b).

As we all know, traditional method used for the production of glucose from starch usually requires two steps: liquefaction via thermostable neutral-α-amylase at pH 6.0 and 80-115 ℃ for dextrin production and saccharification by glucoamylase at pH 4.2-4.5 and 60-70 ℃ for glucose production from dextrin [23]. pH and temperature changes from the liquefaction step to saccharification step result in the high cost in two-step enzymatic hydrolysis of starch [19]. Previous studies focused mostly on the neutral α-amylases in liquefaction step, while acid α-amylase was not investigated much for liquefaction step [13, 22, 35]. Because acid α-amylase has comparable reaction condition with glucoamylase, the replacement of neutral amylase by acid α-amylase can change the two-step procedure to one step (simultaneously liquefaction and saccharification in one pot) which leads to the reduction of the time and the cost. Usually, glucoamylases used in industry are glycosylated which can enhance the enzyme stability [36]. Hence, changing the ratio of low-glycosylated and high-glycosylated glucoamylases seems to be a feasible solution to enhance the total glucoamylase activity during the whole process of saccharification. As described, changing the ratio of O1-2:O1-4 from 10:80 to 40:50 leads to the increase of the reducing sugar by 8.8-16.6%. However, many factors affect glucoamylase glycosylation in vivo, therefore it is difficult to control the ratio of glucoamylase at different glycosylation levels [37, 38].

The total extracellular protein levels in the fermentation cultures of AA-OE and NA-OE strains did not show obvious increase compared with that of the strain O1, but the protein expressions of acid α-amylase and α-amylase are enhanced (Fig. 5a, d). Fermentation cultures of NA-KO strains without α-amylase expression show expected decrease of the extracellular protein levels compared with that of the host strain O1 (Fig. 5e). However, fermentation cultures of AA-KO strains without acid α-amylase expression have no obvious change in extracellular protein levels compared with that of host strain O1 (Fig. 5b). Based on the rough ratio of four isolated and purified proteins, extracellular α-amylase and acid α-amylase account for about 10% and 0.1% of the total extracellular protein (Fig. 2a). We speculate the extracellular protein levels of NA-KO strains were affected more than that of the AA-KO strains. The total glucoamylase activity of both AA-OE and NA-OE strains were all improved compared with that of the host strain O1 (Fig. 5c, f). However, the enhancement of enzymatic activity in AA-OE strains were more obvious than NA-OE strains. On the contrary, the total glucoamylase activity of both AA-KO and NA-KO strains decreased significantly (Fig. 5c, f). However, the enhancement of enzymatic activity in AA-OE strains were more obvious than NA-OE strains, which is different from the results of in vitro synergistic studies that NA had higher effect than AA. This might be due to the limitation of the purification methods, and the amount of AA obtained is less than the amount present in the cultures, so it may reduce the role of AA in in vitro synergistic studies. These results prove that α-amylase and acid α-amylase are necessary for maintaining the total glucoamylase activity, and the absence of acid α-amylase or α-amylase results in a remarkable decrease on the total glucoamylase activity. Generally, α-amylase and acid α-amylase together with glucoamylase convert starch into monosaccharides in industry. α-Amylase, acid α-amylase and glucoamylase exert synergistic effects on the hydrolysis of starch. Overexpression of α-amylase and acid α-amylase provides more non-reducing ends for glucoamylase, and then increases the catalytic efficiency of the glucoamylase enzyme system. Through the synergistic study of the multi-enzymes in the glucoamylase enzyme system in vitro, we found the increases of α-amylase from 9 to 23% or acid α-amylase from 0.1 to 8% can obviously enhance the efficiency of starch saccharification. Obvious effects were also observed in the supernatants of α-amylase or acid α-amylase overexpression strains even if without such high increases of these two enzymes. These may be because some less amount components waiting to be identified in supernatants of fermentation that has important synergistic effects with enzymes in glucoamylase enzyme system, although it is not likely all over 78 proteins identified by LC-MS/MS will have positive effect on the total glucoamylases activity, some of them might be, which is worth analyzing further in the future. Generally, increasing the α-amylase especially acid α-amylase expression can significantly improve the total glucoamylase activity.

## Conclusion

In this study, glucoamylase, α-amylase and acid α-amylase were identified as the main saccharifying enzymes in strain O1 for starch hydrolysis. Moreover, the synergistic study revealed that adjusting the ratio of the key saccharifying enzymes could improve the total glucoamylase activity of glucoamylase enzyme system. Furthermore, adjusting the ratio of key saccharifying enzymes secreted from strain O1 by genetic manipulation enhanced the starch saccharification efficiency of the glucoamylase enzyme system.

## Methods

### Strains, media and growth conditions

*Aspergillus niger* glucoamylase-producing industrial strain O1 with aconidial phenotype provided by Longda Biotechnology Inc. (Shandong, China). All the transformants were obtained by protoplast-mediated transformation. Strain O1 and transformants were cultivated at 34 ℃ using Czapek-Dox solid medium. The composition of Czapek-Dox solid medium is as follows (g/L): sucrose (30), NaNO_3_ (2), MgSO_4_·7H_2_O (1.02), KCl (0.5), FeSO_4_·7H_2_O (0.0183), K_2_HPO_4_ (1), agar (15), pH 6.0. Shake-flask medium contains 100 g/L glucose, 30 g/L soybean flour and 30 ml/L corn steep liquor (pH 5.6). Moderate amount of mycelium was inoculated into 50 mL fermentation medium in a 250-mL Erlenmeyer flask. Shake-flask fermentation was performed at 34 ℃, 240 rpm for 6 days. 2-day, 4-day and 6-day culture supernatants were collected and used for secreted proteome, protein concentration and glucoamylase activity analysis. *Escherichia coli* DH5α was used for plasmid proliferation and was cultured at 37 ℃ in Luria-Bertani (LB) medium added with kanamycin or ampicillin (100 mg/L).

### Construction of the plasmids for overexpression and deletion of α-amylase or acid α-amylase gene

The primers used in this study are listed in Additional file 3: Table S1. The α-amylase gene (ANI_1_260044) was amplified using the primers An-amyA-*Spe*I-F/An-amyA-*EcoR*I-R and acid α-amylase gene (ANI_1_460094) was amplified using the primers An-amyB-*Spe*I-F/An-amyB-*EcoR*V-R from *Aspergillus niger* O1 genomic DNA. Then the identified fragments of α-amylase and acid α-amylase gene were, respectively, linked to *Spe*I/*EcoR*I- and *Spe*I/*EcoR*V-digested PAN52-AnPtef-TtrpC vector using T4 DNA ligase to finish the construction of overexpression plasmids.

For the deletion of the α-amylase and acid α-amylase gene, gene deletion substrates were constructed. the 5′ and 3′-flanking sequences of ANI_1_260044 and ANI_1_460094 were amplified using the primers 2amyA-donor-F1/R1, 2amyA-donor-F3/R3 and amyB-donor-F1/R1, amyB-donor-F3/R3, respectively. PtrpC-neo cassette was amplified from P0380-neo using the primers 2amyA-donor-F2/R2 and amyB-donor-F2/R2 for α-amylase and acid α-amylase gene deletion, respectively. The fragment 5′-PtrpC-neo-3′ was integrated by overlapping PCR and cloned into the Pjet1.2/blunt cloning vector to generate donor DNA sequences. Specific sgRNAs targeting α-amylase and acid α-amylase genes were designed using the sgRNACas9 tool and sgRNA target sites with high scores were selected. All protospacer sequences used to target the genes are presented in Additional file 4. U6 promoter was amplified from the genome of *A. niger* O1. sgRNAs and sgRNA scaffold fragment were fused by overlapping PCR. The final resulting fusion fragments were cloned into Pjet1.2/blunt cloning vector for sequencing. The Cas9-expression PCR cassette AnPtef-Cas9-TtrpC was amplified using primers from the plasmid P0380-AnPtef-Cas9-TtrpC constructed in our lab.

### Transformation of *A. niger* protoplasts

Transformation of *A. niger* protoplasts was performed according to a previously described procedure with some modifications [39]. For target gene expression, 10 μg linearized plasmid was added to the fungal protoplasts. For gene disruption by the CRISPR/Cas9 system, 10 μg Cas9-expression cassette AnPtef-Cas9-TtrpC, sgRNA expression cassettes and the corresponding donor fragments were mixed with a molar concentration ratio of 1:1:1 and then added to fungal protoplasts [40]. The transformants were cultivated on MM medium at 34 ℃ for 3 days with selected resistance geneticin (250 mg/L). α-Amylase and acid α-amylase overexpression or disruption strains were confirmed through diagnostic PCR.

### Total RNA extraction and quantitative real-time PCR

The mycelia after 6-day shake-flask cultivation were harvested by vacuum filtration, and then homogenized in liquid nitrogen for total RNA extraction. Total RNA was isolated and purified using a Qiagen RNeasy Mini Kit (Qiagen, Hilden, Germany). Total RNA was synthesized to first strand cDNA using ReverTra Ace qPCR RT Kit (TOYOBO, Osaka, Japan). qRT-PCR was performed with SYBR Green Real-time PCR Master Mix (TOYOBO, Osaka, Japan). The primers used for genes are listed in Additional file 3: Table S1. The *actin* gene (ANI_1_106134) was used as an internal control. The reaction system and reaction condition were carried out as previously described [40]. The expression level of each gene was estimated using the 2^−ΔΔCT^ method [41]. The transcription level in each transformant to that in the O1 strain was calculated as the relative expression level.

### Glucoamylase enzymatic activity assay

The glucoamylase activity was determined by a modified 3,5-dinitrosalicylic acid method using soluble starch as the substrate. Briefly, 25 μL of 8% soluble starch mixed with 25 μL 100 mM citrate-phosphate buffer, pH 5.0. The mixture was incubated at 62 ℃ for 5 min followed by the addition of 50 μL moderately diluted culture supernatant for further 10 min incubation. Subsequently, 150 μL dinitrosalicylic acid was added to stop the reaction. Then, the samples were boiled for 7 min and 1 mL distilled water was added. The glucoamylase activity was subsequently evaluated by measuring the reducing sugars released from starch hydrolysis at 550 nm.

### Secretome analysis by liquid chromatography-tandem mass spectrometry (LC–MS/MS)

*A. niger* O1 was cultured in fermentation medium and the cultures of 2 days, 4 days and 6 days were collected. The cultures were centrifuged and then filtered through a 0.22-μm PES membrane (Millipore). Protein concentrations of the culture supernatants were determined using Bradford assay (Bio-Rad Laboratories, CA) with bovine serum albumin (BSA) as standard according to the manufacturer’s instructions. The supernatants of 2 days, 4 days, 6 days cultures were assayed by SDS-PAGE. Then, the SDS-PAGE gel was cut into small pieces. In-gel protein digestion was performed following the previously published protocol [42]. The LC-MS/MS analysis was performed with an Eksigent Nano LC coupled to Triple-TOF 5600 mass spectrometer (SCIEX, USA) with a nano-electrospray ionization source [43]. The top 40 precursor ions with the most intensity were fragmented with 22 s of dynamic exclusion time. The WIFF files from shotgun data acquisition were searched against the Uniprot database of *Aspergillus niger* (strain CBS 513.88/FGSC A1513, 2020.04, 14084 entries) in MaxQuant (ver. 1.6.3.4) search engine [44]. Trypsin was set as the specific enzyme, and up to two missed cleavages per peptide were allowed. Precursor ion mass tolerance was set to 20 ppm and fragment ion tolerance was 0.05 Da. For protein identification, the peptide false discovery rates (FDR) were set at 1%.

### Isolation, purification, and identification of starch saccharification-related enzymes from the secreted proteins of *A. niger* O1

The filtered supernatant was desalted by Amicon® ultra-centrifugal filter (membrane with cut-off size of 5 kDa). Subsequently, the desalted supernatant was applied to anion exchange column (HiTrap Q HP, 5 mL, GE) previously equilibrated with buffer A (20 mM potassium phosphate, pH 7.0). The proteins that bind to the column were firstly washed with 0% buffer B (20 mM potassium phosphate, 250 mM NaCl, pH 7.0) at a flow rate of 2 mL/min to remove some impurities. Buffer B was increased from 0 to 100% in 60 min at a flow rate of 3 mL/min to elute proteins and the eluted fractions were collected with 96-well deep-hole plates (1.8 mL/well). The eluted fractions corresponding to each UV peak were monitored using SDS-PAGE. The fractions corresponding to each pure band on SDS-PAGE gel were applied for protein identification using LC-MS/MS. The concentration of purified enzyme was determined by Braford assay kit using BSA as standard. The purified enzyme liquid was frozen and dried into powder and stored at - 80 ℃ for further use.

### Characterization of each component function of glucoamylases cocktail secreted from *A. niger* O1

The activity of each enzyme for real substrate (starch) was determined as follows: 50 μL 100 ng/μL enzyme protein, 25 μL 8% starch (after boiling treatment), 25 μL 100 mM citrate phosphate buffer (pH 5.0). After incubation at 60 ℃ for 10 min, the yield of reducing sugar was measured by DNS method [45]. The optimal pH of each enzyme was measured in 100 mM citrate phosphate buffer with different pH (2.2, 3.0, 4.0, 4.2, 4.4, 4.6, 4.8, 5.0, 6.0, 7.0, and 8.0). The optimal reaction temperature of each enzyme was measured at different temperatures (50, 60, 62, 64, 66, 68, 70, 80, 90 ℃) and pH 5.0. Deglycosylation of O1-2 and O1-4 was conducted with PNGase F (peptide-*N*-Glycosidase F) as described in the manual instruction.

### Synergistic effects of the multiple saccharifying enzymes on starch saccharification

The synergistic reactions of multiple saccharifying enzymes were carried out at 62 ℃ for 4 and 8 h, and the reducing sugars produced were measured using DNS assay. In order to monitor the effect of neutral α-amylase in synergistic system on starch saccharification, different concentrations of neutral α-amylase (0, 112.5, 225, 337.5, 562.5, 1125 ng/mL) were added in the reaction system containing 10% starch, 125 ng/mL low-glycosylated glucoamylase, 1000 ng/mL high-glycosylated glucoamylase and 1.25 ng/mL acid α-amylase in 100 mM citrate phosphate buffer (pH 5.0). For detecting the effect of acid α-amylase in synergistic system on starch saccharification, different concentrations of acid α-amylase (0, 1.25, 6.25, 12.5, 112.5, 225, 337.5 ng/mL) were added in the reaction system containing 10% starch, 125 ng/mL low-glycosylated glucoamylase, 1000 ng/mL high-glycosylated glucoamylase and 125 ng/mL neutral α-amylase in 100 mM citrate phosphate buffer (pH 5.0). The optimum ratio of two glucoamylases was also measured. The reaction system contained 10% starch, 50 ng/mL neutral α-amylase, 5 ng/mL acid α-amylase and 4500 ng/mL total glucoamylase with different ratios (1:89, 5:85, 10:80, 20:70, 30:60, 4050, 50:40, 60:30, 70:20, 80:10, 90:0) in 100 mM citrate phosphate buffer (pH 5.0).

## Supplementary Information

**Additional file 1: Figure S1.** Glycoside hydrolases and relative abundance detected in 2d, 4d and 6d fermentation supernatants.

**Additional file 2.** The results of the peptide fragments of O1-1, O1-2, O1-3 and O1-4 searched against the Uniprot database of *Aspergillus niger*.

**Additional file 3: Table S1.** List of PCR primers used in this study.

**Additional file 4.** Nucleotide sequences of the sgRNA expression cassettes.

**Additional file 5: Figure S2.** Electrophoretic analysis of purified O1-2 and O1-4 with or without digestion with peptide-N-glycosidase F. 1. Before deglycosylation of O-4, 2. After deglycosylation of O-4, 3. Before deglycosylation of O-2, 4. After deglycosylation of O-2.

**Additional file 6: Figure S3.** Relative transcription level of acid α-amylase (AA) and α-amylase (NA) genes in O1 strain and transformants. a Relative transcription level of AA in stain O1 and AA-overexpression and AA knock out transformants. b Relative transcription level of NA in stain O1 and NA-overexpression and NA knock out transformants.

## Data Availability

All the supporting data are available.
