# Supplementary figures and images for: Synergistic effects of multiple enzymes from industrial Aspergillus niger strain O1 on starch saccharification
Source: Biotechnol Biofuels. 2021 Nov 27;14:225. doi: 10.1186/s13068-021-02074-x (PMC8627030; doi:10.1186/s13068-021-02074-x)

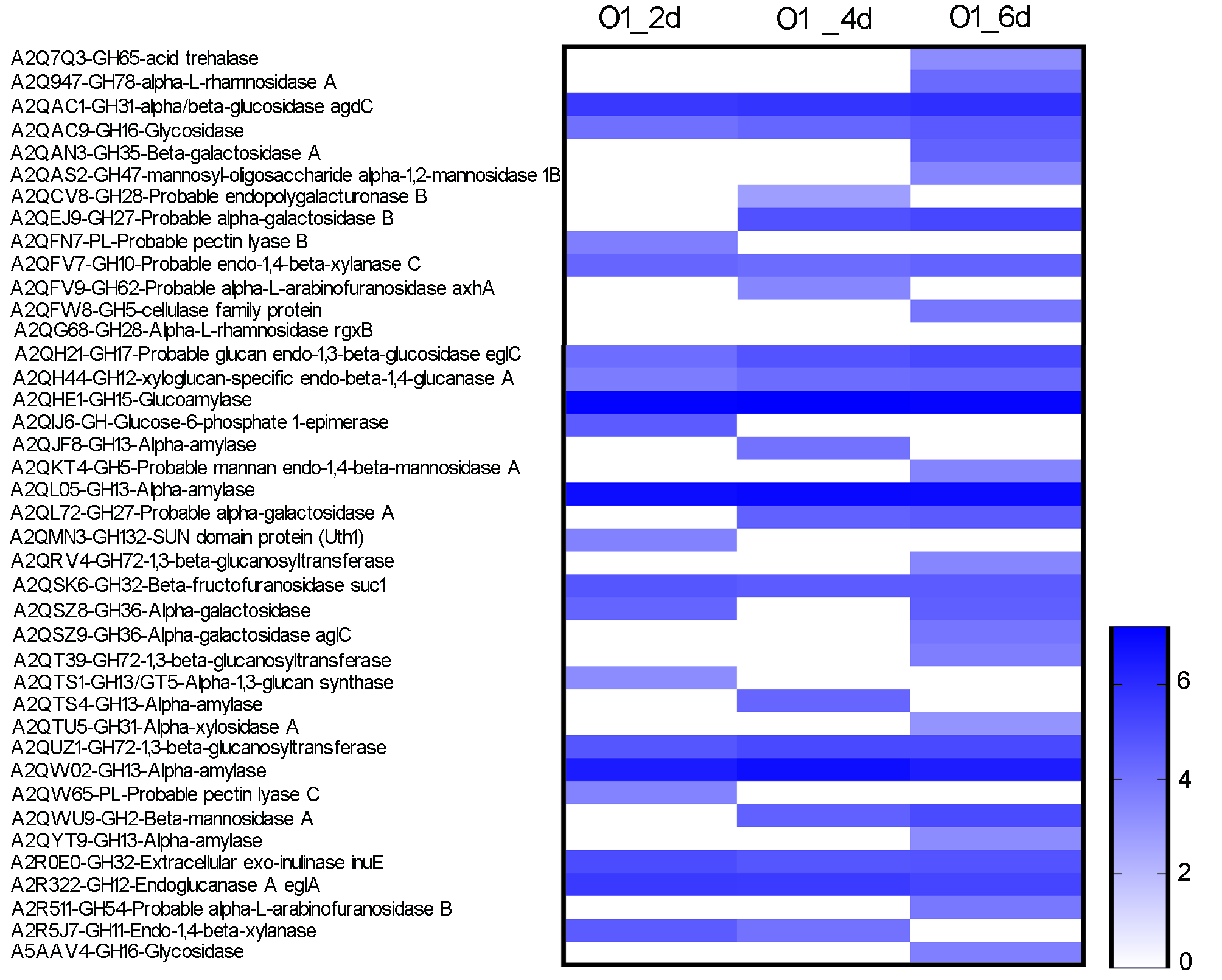

Supplement: Supplementary file 1 — Additional file 1: Figure S1. Glycoside hydrolases and relative abundance detected in 2d, 4d and 6d fermentation supernatants. [file 13068_2021_2074_MOESM1_ESM.tif]

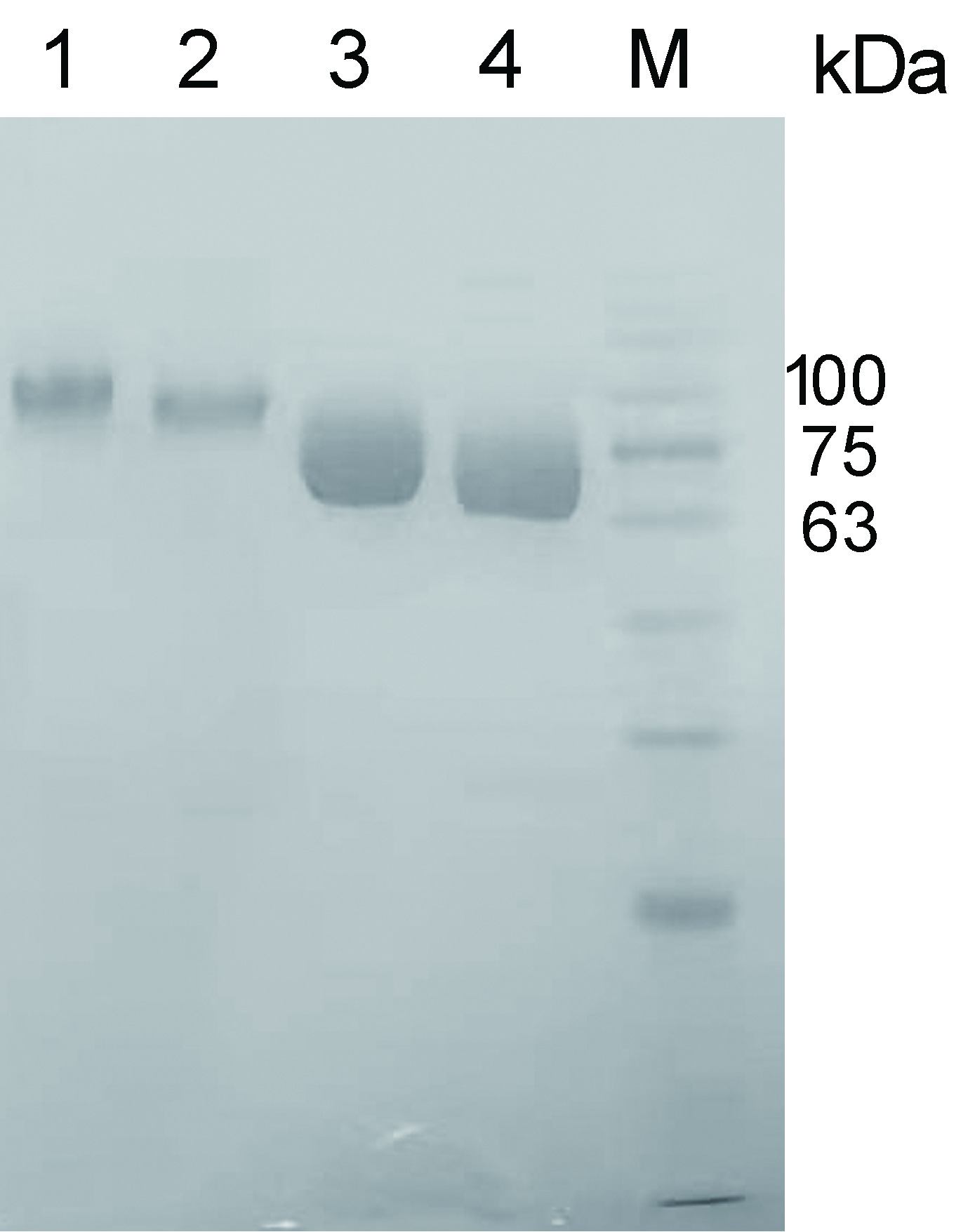

Supplement: Supplementary file 5 — Additional file 5: Figure S2. Electrophoretic analysis of purified O1-2 and O1-4 with or without digestion with peptide-N-glycosidase F. 1. Before deglycosylation of O-4, 2. After deglycosylation of O-4, 3. Before deglycosylation of O-2, 4. After deglycosylation of O-2. [file 13068_2021_2074_MOESM5_ESM.tif]

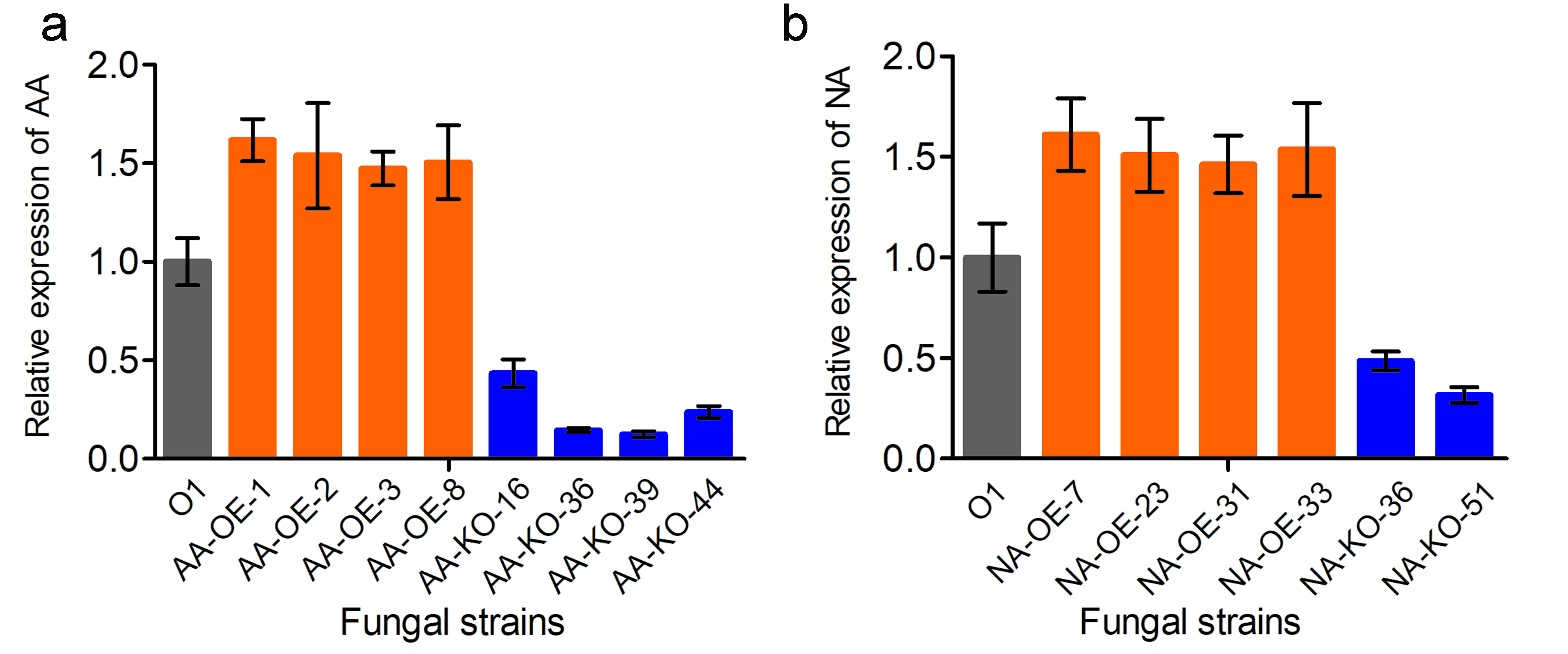

Supplement: Supplementary file 6 — Additional file 6: Figure S3. Relative transcription level of acid α-amylase (AA) and α-amylase (NA) genes in O1 strain and transformants. a Relative transcription level of AA in stain O1 and AA-overexpression and AA knock out transformants. b Relative transcription level of NA in stain O1 and NA-overexpression and NA knock out transformants. [file 13068_2021_2074_MOESM6_ESM.tif]
