# Supplementary material for: Synergistic effects of multiple enzymes from industrial Aspergillus niger strain O1 on starch saccharification
Source: Biotechnol Biofuels. 2021 Nov 27;14:225. doi: 10.1186/s13068-021-02074-x (PMC8627030; doi:10.1186/s13068-021-02074-x)
Supplement: Supplementary file 3 — Additional file 3: Table S1. List of PCR primers used in this study. [file 13068_2021_2074_MOESM3_ESM.docx]

| Cloning of α-amylase gene | An-amyA-SpeI-F | ACTAGTATGATGGTCGCGTGGTGGTC |
| --- | --- | --- |
| Cloning of α-amylase gene | An-amyA-EcoRI-R | GAATTCTCACGAGCTACTACAGATCTTGC |
| Cloning of acid α-amylase gene | An-amyB-Spe-F | ACTAGTATGAGATTATCGACTTCGAGTCTC |
| Cloning of acid α-amylase gene | An-amyB-EcoRV-R | GATATCTTACTCGACGTATAATCTTCCGCT |
| Cloning 5’ of α-amylase gene | 2amyA-donor-F1 | ATGATGGTCGCGTGGTGGTCTCTATTTCTGTACG |
| Cloning 5’ of α-amylase gene | 2amyA-donor-R1 | CTTCAATATCAGTTAACGTCGCGTACCATTCATTCTTGACCAC |
| Cloning 3’ of α-amylase gene | 2amyA-donor-F3 | CTTCTTGACGAGTTCTTCTGACGGGTACAACAAAGCCGCAGG |
| Cloning 3’ of α-amylase gene | 2amyA-donor-R3 | TCACGAGCTACTACAGATCTTGCTACCTGCCAACTTC |
| Cloning 5’ of acid α-amylase gene | amyB-donor-F1 | GGAACCAGTACGGCAGCTGATAGTATCCGAAAG |
| Cloning 5’ of acid α-amylase gene | amyB-donor-R1 | CTCCTTCAATATCAGTTAACGTCGCGAACCGATCCGTCAATAGG |
| Cloning 3’ of acid α-amylase gene | amyB-donor-F3 | CTTCTTGACGAGTTCTTCTGATGGATCTCGCCTATCACTGAAC |
| Cloning 3’ of acid α-amylase gene | amyB-donor-R3 | GTTCGTGGTGGCTATCCAGGTGTACAGCTCTG |
| Cloning of *neo* gene | 2amyA-donor-F2 | GTGGTCAAGAATGAATGGTACGCGACGTTAACTGATATTGAAG |
| Cloning of *neo* gene | 2amyA-donor-R2 | CCTGCGGCTTTGTTGTACCCGTCAGAAGAACTCGTCAAGAAG |
| Cloning of *neo* gene | amyB-donor-F2 | CCTATTGACGGATCGGTTCGCGACGTTAACTGATATTGAAGGAG |
| Cloning of *neo* gene | amyB-donor-R2 | GTTCAGTGATAGGCGAGATCCATCAGAAGAACTCGTCAAGAAG |
| Cloning of *cas9* gene | An Ptef/cas9-F | CTGGTACGGTACCAAATCTTG |
| Cloning of *cas9* gene | An Ptef/cas9-R | AAGAAGGATTACCTCTAAAC |
| Diagnostic PCR for α-amylase deletion | 2amyA-KO-F | GCTTCACAGCCATCTGGATC |
| Diagnostic PCR for α-amylase deletion | 2amyA-KO-R | CTTGTACAGCTCGCTGTCGGT |
| Diagnostic PCR for acid α-amylase deletion | amyB-KO-F | GCAGCGATGAGATTATCGAC |
| Diagnostic PCR for acidα-amylase deletion | amyB-KO-R | CACGTCGTATCTGTCAAGTC |
| Quantitative RT-PCR for actin | Actin-RT-F | CGAACTGGGATGACATGGAG |
| Quantitative RT-PCR for actin | Actin-RT-R | ACACCGTCACCAGAGTCCAG |
| Quantitative RT-PCR for acid α-amylase gene | AA-RT-F | CTCCTCCTACTTCCACCCATACTG |
| Quantitative RT-PCR for acid α-amylase gene | AA-RT-R | TAGACACCTGCTGCTTCCTGGTAG |
| Quantitative RT-PCR for α-amylase gene | NA-RT-F | CGGGTAGCTCAGTCGATTACAGTG |
| Quantitative RT-PCR for α-amylase gene | NA-RT-R | CCGTCAATGGAGTAGTTCGATACC |

Additional file 3: Table S1 List of PCR primers used in this study.
