# Supplementary material for: Synergistic effects of multiple enzymes from industrial Aspergillus niger strain O1 on starch saccharification
Source: Biotechnol Biofuels. 2021 Nov 27;14:225. doi: 10.1186/s13068-021-02074-x (PMC8627030; doi:10.1186/s13068-021-02074-x)
Supplement: Supplementary file 4 — Additional file 4. Nucleotide sequences of the sgRNA expression cassettes. [file 13068_2021_2074_MOESM4_ESM.docx]

Additional file 4: Nucleotide sequence of the sgRNA expression cassettes. Blue letters indicate

the U6 promoter. Orange letters indicate the sgRNA scaffold. red letters indicate the target sequence of α-amylase and acid α-amylase gene, respectively.

>2amyA-sgRNA-cassette-1

Cagttacttataagcttggagcttggatctctttgaggtggaccttccttgaagggtttcatctctgtactatcatgcgaatgctaaagcagaactttaacagaaccaccagtgtctaataaattcgatccgtatattgtgcaccattactcatctgtgtttcccccaaacatgcagtctcctgcgcagatagactgtcaactatagtaattcccgtccgcgaagccgccctatccaaaagtgtattaccctctcttgtatgcaacaagagtcgttctttctcgcgctaatacccatccgtctatcgcacaattaaaccttctgatccctacaatttgcctgacaaaataaatgaagttcaacgtgcaaacaagctagagccagtgtacattgagtatcatctgcagctctactcaaggtactatagtacctcagccaatttgatgttcctgccttcccgcccctcgcttagccgaccaattagagttcgttaattctaaccattattcctatataattcaaagaatgaatggtacgactggggttttagagctagaaatagcaagttaaaataaggctagtccgttatcaacttgaaaaagtggcaccgagtcggtgctttttt

>2amyA-sgRNA-cassette-2

cagttacttataagcttggagcttggatctctttgaggtggaccttccttgaagggtttcatctctgtactatcatgcgaatgctaaagcagaactttaacagaaccaccagtgtctaataaattcgatccgtatattgtgcaccattactcatctgtgtttcccccaaacatgcagtctcctgcgcagatagactgtcaactatagtaattcccgtccgcgaagccgccctatccaaaagtgtattaccctctcttgtatgcaacaagagtcgttctttctcgcgctaatacccatccgtctatcgcacaattaaaccttctgatccctacaatttgcctgacaaaataaatgaagttcaacgtgcaaacaagctagagccagtgtacattgagtatcatctgcagctctactcaaggtactatagtacctcagccaatttgatgttcctgccttcccgcccctcgcttagccgaccaattagagttcgttaattctaaccattattcctatataattcaaagtccagaaggacttctggccgttttagagctagaaatagcaagttaaaataaggctagtccgttatcaacttgaaaaagtggcaccgagtcggtgctttttt

>amyB-sgRNA-cassette-1

Cagttacttataagcttggagcttggatctctttgaggtggaccttccttgaagggtttcatctctgtactatcatgcgaatgctaaagcagaactttaacagaaccaccagtgtctaataaattcgatccgtatattgtgcaccattactcatctgtgtttcccccaaacatgcagtctcctgcgcagatagactgtcaactatagtaattcccgtccgcgaagccgccctatccaaaagtgtattaccctctcttgtatgcaacaagagtcgttctttctcgcgctaatacccatccgtctatcgcacaattaaaccttctgatccctacaatttgcctgacaaaataaatgaagttcaacgtgcaaacaagctagagccagtgtacattgagtatcatctgcagctctactcaaggtactatagtacctcagccaatttgatgttcctgccttcccgcccctcgcttagccgaccaattagagttcgttaattctaaccattattcctatataattcaaagacggatcggttcggtaggagttttagagctagaaatagcaagttaaaataaggctagtccgttatcaacttgaaaaagtggcaccgagtcggtgctttttt

>amyB-sgRNA-cassette-2

Cagttacttataagcttggagcttggatctctttgaggtggaccttccttgaagggtttcatctctgtactatcatgcgaatgctaaagcagaactttaacagaaccaccagtgtctaataaattcgatccgtatattgtgcaccattactcatctgtgtttcccccaaacatgcagtctcctgcgcagatagactgtcaactatagtaattcccgtccgcgaagccgccctatccaaaagtgtattaccctctcttgtatgcaacaagagtcgttctttctcgcgctaatacccatccgtctatcgcacaattaaaccttctgatccctacaatttgcctgacaaaataaatgaagttcaacgtgcaaacaagctagagccagtgtacattgagtatcatctgcagctctactcaaggtactatagtacctcagccaatttgatgttcctgccttcccgcccctcgcttagccgaccaattagagttcgttaattctaaccattattcctatataattcaaagcatgggattcacggccatcgttttagagctagaaatagcaagttaaaataaggctagtccgttatcaacttgaaaaagtggcaccgagtcggtgctttttt
